# TLR4-NLRP3-GSDMD-Mediated Pyroptosis Plays an Important Role in Aggravated Liver Injury of CD38^−/−^ Sepsis Mice

**DOI:** 10.1155/2021/6687555

**Published:** 2021-03-30

**Authors:** Huiqing Zhang, Yuna Du, Yujie Guo, Zeyu Wang, Hua Li, Zhe Lv, Lifeng Zeng, Yiguo Chen, Zhengyu Xie, Rong Li

**Affiliations:** ^1^Department of Clinical Laboratory, Jiangxi Provincial People's Hospital and Affiliated People's Hospital of Nanchang University, Nanchang, China; ^2^Department of Clinical Laboratory, The Second Affiliated Hospital of Nanchang University & Jiangxi Provincial Key Laboratory for Laboratory Medicine, Nanchang, China

## Abstract

Clinically, severe bacterial infection can cause septicemia and multiple organ dysfunction syndrome, especially liver injury. CD38 is closely related to many inflammatory pathways, but its role in liver injury caused by bacterial infection remains unclear. The purpose of this study is to discuss the specific role of CD38 in bacterial liver injury. Eight-week-old male C57BL/6 mice (WT, CD38^−/−^ and CD38^−/−^TLR4^mut^) were used and stimulated with *Escherichia coli* (ATCC25922) or PBS, intraperitoneally. After 3 hours of bacterial stimulation, serum was collected to detect ALT and AST concentration, and liver tissue was harvested for hematoxylin and eosin staining and bacterial culture. The mRNA expressions of TLR4, NLRP3, IL-1*β*, IL-18, and GSDMD were quantitatively determined by RT-qPCR. The expressions of TLR4, MyD88, TRIF, NF-*κ*B p65, NLRP3, GSDMD, and cytokines were detected by Western blot. The expression and localization of ERK1/2 were detected by immunohistochemistry and Western blot. The results showed that bacterial stimulation could upregulate the expression of inflammatory cytokines, leading to hepatic dysfunction. Moreover, bacterial stimulation of CD38-deficient mice can aggravate the inflammatory response, the expressions of TLR4, NF-*κ*B, and ERK1/2 were significantly increased, and the biomarkers related to pyroptosis also manifested more obvious pyroptosis. However, TLR4 mutation significantly alleviated inflammation and pyroptosis in the liver caused by bacteria, on the basis of CD38 deficiency. Overall, CD38 knockout exacerbates bacteria-induced liver damage through TLR4-NLRP3-GSDMD-mediated pyroptosis.

## 1. Introduction

Clinically, severe bacterial infection can cause septicemia, and sepsis has been recognized as a global health priority by WHO because of the current estimates of 30 million episodes and six million deaths per year [1]. And the life-threatening organ dysfunction caused by dysregulated host response to infection is a thorny issue in sepsis [2], including liver injury, one of the main clinical manifestations and an independent risk factor for multiple organ dysfunction syndrome (MODS) and high mortality rate in patients with sepsis [3]. Hepatic dysfunction is considered to be one component of the MODS and usually associated with a poor prognosis in clinic during sepsis [4]. Moreover, the study has shown that liver injury due to sepsis was shown to be induced via oxidative damage, inflammatory response, and neutrophil infiltration [5] and hepatic pathology in sepsis mainly includes steatosis, cholangiolitis, intrahepatic cholestasis, periportal inflammation, and apoptosis [6].

Nicotinamide adenine dinucleotide (NAD^+^) is a coenzyme in energy metabolism and electron transfer and regulates energy metabolism, mitochondrial function, apoptosis, necrosis, nuclear gene expression, and neurotransmitter release [7]. Cluster of differentiation 38 (CD38), a type II transmembrane protein, is the major ADP-ribosyl cyclase in mammals, which is a key NAD^+^-dependent enzyme [8]. CD38 is a multifunctional transmembrane protein that is widely expressed in immune cells [9], controls the innate immune response against infection [10, 11], and closely relates to inflammatory pathways such as the Toll-like receptor (TLR) pathway [12, 13] and mitogen-activated protein kinase (MAPK) pathway [13, 14]. CD38 also plays a critical role in many liver diseases such as glucagon-induced gluconeogenesis in hepatocytes [15] and lipopolysaccharide- (LPS-) induced acute damage of the liver [16]. And in inflammatory liver disease, the CD38/NAADP-mediated Ca^2+^ signaling pathway is a potential novel therapeutic target [17]. Moreover, it has been reported that CD38 is expressed in inflammatory cells [18], regulates the function of immunocytes [19], depresses the expressions of inflammatory cytokines, and inhibits the death of hepatocytes [16].

The innate immune system is considered to be the main defense against invading pathogens and maintaining homeostasis. The pathogens, damaged tissues, and cells may release pathogen-related molecular patterns (PAMPs) and injury-related molecular patterns (DAMPs). Then, pattern recognition receptors (PRRs) will recognize them and activate immune response. Among these receptors, NLR family protein 3 (NLRP3) is the most well-studied Nod-like receptor, because NLRs may form a protein complex called inflammasome [20]. The inflammasome is a multiprotein complex that is composed of NLRP3, apoptosis-associated speck-like protein (ASC), and serine protease caspase-1 (caspase-1), mainly mediating the host's immune response to microbial infection and cell damage [21]. The aggregation of the inflammasome leads to the cleavage of procaspase-1 and the formation of activated caspase-1. Cleaved caspase-1 promotes the transformation of prointerleukin-1 beta (pro-IL-1*β*) and prointerleukin-18 (pro-IL-18) into mature IL-1*β* and IL-18 [22]. In many immune responses, mature IL-1*β* is an effective proinflammatory mediator, which can recruit innate immune cells to the infection site and regulate acquired immune cells, while mature IL-18 can promote expression of interferon gamma (IFN-*γ*). At the same time, activated caspase-1 can cleave gasdermin-D (GSDMD) and induce proinflammatory cell death called pyroptosis [23, 24]. More and more studies have shown that NLRP3-meditated pyroptosis may cause vital injury in different organs affected by sepsis including liver injury [21, 25, 26]. And pyroptosis was found not only in immunocytes such as monocytes or macrophages but also in hepatocytes [21, 24]. Up to now, some mechanism studies [27, 28] have shown that inhibiting the assembly and activation of the inflammasome can improve the proinflammatory response in sepsis. However, the mechanism and effect of the NLRP3 inflammasome on the pathophysiology of sepsis need further study.

Recently, some study reported that Toll-like receptor 4 (TLR4) is a therapeutic target for the prevention and treatment of liver failure [29]. TLR4 is an important member of the Toll-like receptor family and can activate downstream intracellular signals such as nuclear factor-*κ*B (NF-*κ*B) and MAPK after LPS recognition [30, 31]. And the activated NF-*κ*B and MAPKs pathways in turn lead to further inflammation responses, more production of proinflammatory cytokines, or immunosuppression [5, 32]. In addition, activated NF-*κ*B can induce nitric oxide (NO) production and activate apoptotic protein to induce apoptosis and necrosis, and the final outcome is organ failure [5]. Furthermore, the extracellular signal-regulated kinase 1/2 (ERK1/2) signaling pathway, one of the MAPK superfamily pathways, is known to play an important role in the regulation of inflammation [33, 34] and apoptosis [35–37]. And it has been confirmed by researchers that the ERK1/2 signaling pathway plays a key role in liver injury [34, 36, 38].

Our previous studies have shown that TLR4 expression increases noticeably in CD38^−/−^ mice when compared to wild-type (WT) mice. Therefore, in order to further explore the role of CD38 in the TLR4-related pyroptosis-meditated inflammatory signaling pathway and the potential role of CD38 in liver injury caused by bacterial infection, we injected *E. coli* into the abdominal cavity of mice (WT, CD38^−/−^, and CD38^−/−^TLR4^mut^) and then observed the pathological changes in liver tissues, expression variation of liver function indicator, cytokines, biomarkers of pyroptosis, and signaling pathways to reveal the effect of CD38 deficiency on bacterial infection-mediated sepsis liver injury.

## 2. Materials and Methods

### 2.1. Animals and Establishment of the Mouse Model

Wild-type mice (C57BL/6 of 4 weeks old) were purchased from the Laboratory Animal Center of Wuhan University, and C57BL/6 background CD38^−/−^ and CD38^−/−^TLR4^mut^ mice (B6.129P2-Ly96^−/−^) were kindly donated by professors Hongbo Xin and Keyu Deng from the Translational Medicine Research Institute of Nanchang University. All groups of mice were raised in SPF Animal Facility at the laboratory animal center of Nanchang University and kept well fed and watered. Eight-week-old male mice (20 ± 2 g) were selected for the experiment. All experiments were in conformance with the Management Ordinance of Laboratory Animals of Nanchang University and were approved by the Animal Protection Committee of Nanchang University.

A standard *Escherichia coli* (*E. coli*) strain (ATCC25922) was used to establish mouse models of sepsis, which was kindly offered by the Laboratory of Jiangxi Provincial People's Hospital. A ring (2 *μ*l) of resoluble bacterial solution was added into Columbia blood plate medium with three-zone scribing and then cultured in an incubator with atmosphere at 37°C for 12 hours. Intraperitoneal injection of 0.5 ml *E. coli* (3 × 10^8^ cfu/ml) was used to induce sepsis according to a research by Shen et al. [39], and equal amounts of PBS were injected as a control. Three hours later, mice were sacrificed, and the blood and liver were collected for further analysis.

### 2.2. Serum Analysis

Blood was collected three hours after *E. coli* or PBS injection. Then, the supernatant was obtained after centrifugation at 10621g at 4°C for 10 minutes. And the concentrations of serum aspartate aminotransferase (AST) and alanine aminotransferase (ALT) were determined by using ELISA kits (Nanjing Jiancheng Bioengineering Institute, China) according to the instructions.

### 2.3. Bacterial Culture of Liver Tissue and Gram Staining

A total of 100 mg of liver tissue was taken into the homogenizer and then ground completely. And the homogenates (100 *μ*l) were applied to agar plates and distributed evenly. The plates were cultured in a 5% CO_2_ incubator at 37°C for 24 hours and screened for bacterial colonies. A single colony was selected and transferred onto a slide. The slides were Gram stained and observed under the microscope.

### 2.4. Hematoxylin and Eosin Staining

Three hours after *E. coli* or PBS injection, the mice were killed and livers were taken out. Then, the tissues were fixed in 4% paraformaldehyde solution overnight and washed with 0.1 M phosphate buffer (pH 7.4) for 10 minutes 3 times. The liver tissues were rendered transparent with xylene and embedded after being dehydrated by successive washes in an ethanol gradient (60%, 75%, 85%, 95% twice, and 100% twice). Paraffin sections of 3 *μ*m were prepared using a microtome. The sections were heated at 65°C for 2 hours for deparaffinization and hydrated by submersion in the following solutions: xylene for 5 minutes three times, 100% ethanol for 30 seconds twice, and 95% ethanol and 70% ethanol for 30 seconds. Hematoxylin and eosin staining (H&E) was performed, and the morphological changes were evaluated under a microscope.

### 2.5. RNA Extraction and Real-Time Quantitative PCR Assay

Total RNA was extracted from liver tissues (50-100 mg) with the TRizol reagent (Invitrogen, US). And then, the extracted RNA (1 *μ*g) was treated with gDNA Eraser (Takara Biotech, Japan) at 42°C for 2 minutes to remove the genomic DNA. RT-PCR was performed using PrimeScript™ RT under the following conditions: 37°C for 15 minutes, 85°C for 5 seconds, and 4°C indefinitely for product preservation.

RT-qPCR was performed using SYBR® Premix Ex Taq™ II (Tli RNaseH Plus) (Takara Biotech, Japan) and the StepOne™ PLUS Real-Time PCR System (Applied Biosystems, New York, USA). We used primers for TLR4, NLRP3, IL-1*β*, IL-18, GSDMD, and GAPDH synthesized from TSINGKE Biotech (TSINGKE Biotech, Beijing, China) (TLR4 forward primer: 5′-CGCTTTCA CCTCTGCCTTCAC-3′; TLR4 reverse primer: 5′-TTGCCGTTCTTG TTCTTCTTC-3′; NLRP3 forward primer: 5′-GCCGTCTACGTCTTCTTCCTT TCC-3′; NLRP3 reverse primer: 5′-CATCCGCAGCCAGTGAACAGAG-3′; IL-1*β* forward primer: 5′-TTTTCCTCCTTGCCTCTGAT-3′; IL-1*β* reverse primer: 5′-GAGTGCTGCCTAATGTCCCC-3′; IL-18 forward primer: 5′-GACTCTTGC GTCAACTTCAAGG-3′; IL-18 reverse primer: 5′-CAGGCTGTCTTTTGTCAA CGA-3′; GSDMD forward primer: 5′-CCATCGGCCTTTGAGAAAGTG-3′; GSDMD reverse primer: 5′-ACACATGAATAACGGGGTTTCC-3′; GADPH forward primer: 5′-GAAGGTGGTGAAGCAGGCATC; and GADPH reverse primer: 5′-GTGGGAGTTGCTGTT GAAGTC). The PCR program was 95°C for 30 seconds, 40 cycles of 95°C for 5 seconds, and 60°C for 30 seconds. We detected the threshold cycle (Ct) for all genes and determined their relative expression levels compared to GAPDH.

### 2.6. Immunohistochemical Staining

Sections of 3 *μ*m were prepared using a microtome and baked in an oven at 65°C overnight and then dewaxed in xylene for 5 minutes three times, rehydrated in an ethanol gradient, and blocked by incubation in 0.3% H_2_O_2_ for 10 minutes. The slides were incubated with primary antibodies, anti-ERK1/2 rabbit mAb (1 : 250) (CST, USA) for 50 minutes at room temperature, and then with biotin-conjugated secondary antibodies for 25 minutes at room temperature after a thorough wash in PBS three times. The signal was detected with the help of diaminobenzidine (DAB). The samples were fixed on glass slides and observed under the microscope.

### 2.7. Western Blot

Western blot was performed to further detect the protein expressions of TLR4, TRIF, MyD88, NF-*κ*B, IL-6, iNOS, BAX, NLRP3, ASC, caspase-1, IL-1*β*, IL-18, caspase-3, GSDMD, and ERK1/2 in livers from WT, CD38^−/−^, and CD38^−/−^TLR4^mut^ mice (*n* = 3/group). The livers were ground up and lysed in the RIPA Lysis Buffer with phenylmethanesulfonyl fluoride. The equivalent protein sample was loaded into 10% SDS-PAGE gel and transferred onto polyvinylidene fluoride (PVDF) membranes. The primary antibodies anti-TLR4 rabbit mAb (1 : 500) (CST, USA), anti-TRIF rabbit mAb (1 : 1000) (Proteintech, USA), anti-MyD88 mouse mAb (1 : 2000) (Proteintech, USA), anti-NF-*κ*B p65 rabbit mAb (1 : 1000) (CST, USA), anti-phospho-NF-*κ*B p65 rabbit mAb (1 : 500) (CST, USA), anti-IL-6 mouse mAb (1 : 2000) (Proteintech, USA), anti-iNOS rabbit mAb (1 : 1000) (CST, USA), anti-BAX rabbit mAb (1 : 5000) (Proteintech, USA), anti-NLRP3 rabbit mAb (1 : 500) (Boster, China), anti-ASC rabbit mAb (1 : 500) (Affinity, China), anti-caspase-1 rabbit mAb (1 : 500) (Abcam, UK), anti-IL-1*β* rabbit mAb (1 : 1000) (CST, USA), anti-IL-18 rabbit mAb (1 : 1000) (Affinity, China), anti-caspase-3 rabbit mAb (1 : 500) (CST, USA), anti-GSDMD rabbit mAb (1 : 500) (Affinity, China), anti-ERK1/2 rabbit mAb (1 : 1000) (CST, USA), anti-phospho-ERK1/2 rabbit mAb (1 : 2000) (CST, USA), and anti-GAPDH rabbit mAb (1 : 5000) (Proteintech, USA) were used. The expressions of TLR4, TRIF, MyD88, NF-*κ*B, IL-6, iNOS, BAX, NLRP3, ASC, caspase-1, IL-1*β*, IL-18, caspase-3, GSDMD, and ERK1/2 and GAPDH were visualized by the ECL assay (Sage creation) according to the manufacturer's instructions.

### 2.8. Statistical Analysis

All data are depicted as the mean ± standard deviation (SD). A paired *t*-test was used to determine statistically significant differences between two groups, and one-way ANOVA followed by the Tukey post hoc test was used to estimate differences for multigroups. Differences were considered significant if the *p* value is <0.05. All analyses were made with the statistical software GraphPad Pro 5.0 (GraphPad, San Diego, CA, USA).

## 3. Results

### 3.1. *E. coli* Induces Inflammation Leading to Liver Injury in WT Mice

**Figure 1:**
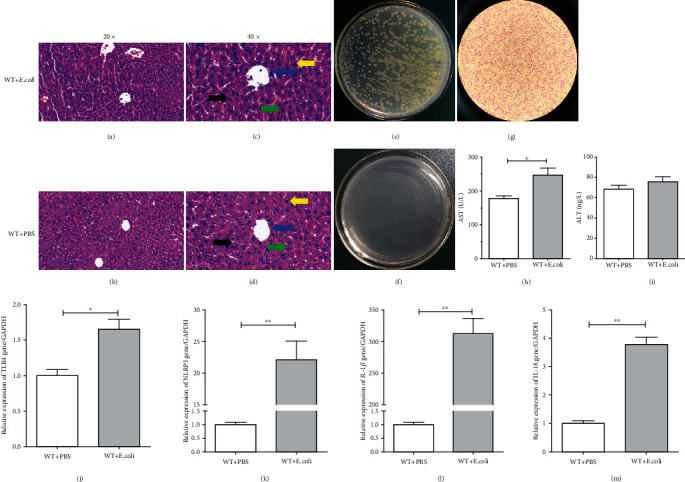
*Escherichia coli* can induce liver injury in mice. WT mice were injected with PBS or 3 × 10^8^ cfu/ml*E. coli* intraperitoneally and sacrificed 3 hours later. Liver pathological injuries were observed with hematoxylin and eosin staining (a–d). The yellow arrows indicate edema, the blue arrows indicate inflammatory cell infiltration, the black arrows indicate punctate necrosis, and the green arrows indicate binucleate hepatocytes. Bacteria from the liver of PBS- or *E. coli*-stimulated WT mice were cultured in MH medium (e, f) overnight and identified by Gram staining (g). The serum AST (h) and ALT (i) concentrations were determined by using the detection kits. The mRNA of liver inflammatory cytokines TLR4 (j), NLRP3 (k), IL-1*β* (l), and IL-18 (m) of PBS- or 3 × 10^8^ cfu/ml*E. coli*-stimulated mice were measured by RT-qPCR. Data are presented as means ± standard deviation. Statistical significance was determined by the paired *t*-test (*n* = 3, ^∗^*p* < 0.05, ^∗∗^*p* < 0.01).

In order to induce liver injury, we injected 3 × 10^8^ cfu/ml*Escherichia coli* (*E. coli*) into the abdominal cavity of WT mice, and the PBS was injected as the control. Three hours later, the liver was harvested for further study. H&E staining results showed that compared with the control group, after 3 hours of *E. coli* stimulation, obvious pathological changes can be observed in the liver of *E. coli*-injected mice, and the hepatocyte edema (yellow arrow), inflammatory cell infiltration (blue arrow), punctate necrosis (black arrow), and binucleate hepatocytes (green arrow) could be observed (Figures 1(a)–1(d)). And a study showed that the occurrence of binucleate hepatocytes activated TLR4-mediated signaling [40] and increases with the necro-inflammatory state, and it may be a reactive cell response to liver injury [41]. Furthermore, the liver suspension was taken for bacterial culture and Gram staining. There were scattered colonies in the stimulation group of *E. coli* (Figure 1(e)) and Gram-negative, rod-shaped bacteria in the bacterial staining (Figure 1(g)). No colony formation was observed in the normal control group (Figure 1(f)). Moreover, compared with the control group, the serum AST increased significantly after 3 hours of *E. coli* stimulation (Figure 1(h)). However, there was no significant difference in serum ALT between the two groups (Figure 1(i)). These data suggested that *E. coli* injected intraperitoneally can enter the liver tissues and cause pathological changes in the liver and damage to liver function.

In order to further explore the effect of *E. coli* stimulation on the liver, RNA was extracted from the liver for RT-qPCR. We found that in the liver tissue, compared with the control group, the TLR4 gene expression in the *E. coli*-stimulated group was significantly higher (Figure 1(j)). At the same time, the gene expression of proinflammatory cytokines NLRP3 (Figure 1(k)), IL-1*β* (Figure 1(l)), and IL-18 (Figure 1(m)) increased significantly. These results showed that *E. coli* can intrude into the liver, cause inflammation, and lead to liver damage.

### 3.2. CD38 Knockout Can Aggravate the Liver Damage Caused by *E. coli*

**Figure 2:**
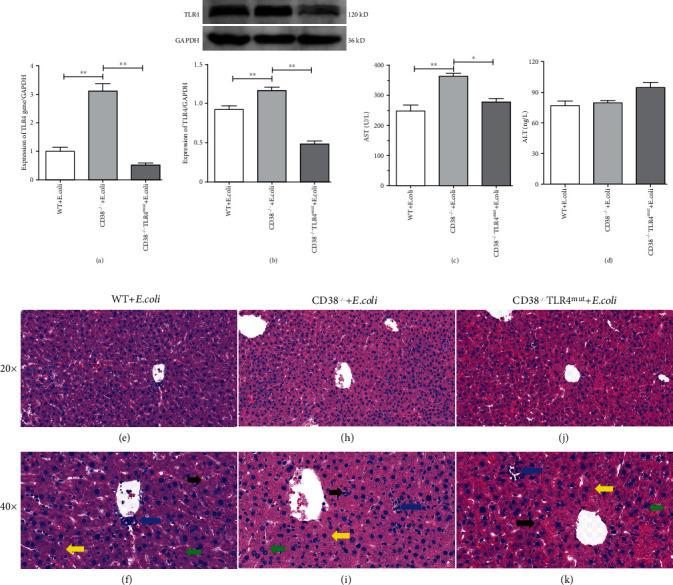
*Escherichia coli* can induce more serious liver injury in CD38^−/−^ mice. The equivalent amount of *E. coli* suspension was injected intraperitoneally into WT, CD38^−/−^, and CD38^−/−^TLR4^mut^ mice, and the liver and serum samples were harvested after 3 hours. The mRNA of TLR4 (a) in the liver was measured by RT-qPCR, and the expression of TLR4 (b) was detected by Western blot. The concentrations of AST (c) and ALT (d) in serum were determined by using the detection kits. And the liver samples were taken for hematoxylin and eosin staining (e, f, h, i, j, k). The yellow arrows indicate edema, the blue arrows indicate inflammatory cell infiltration, the black arrows indicate punctate necrosis, and the green arrows indicate binucleate hepatocytes. Data are presented as means ± standard deviation. Statistical significance was determined by one-way ANOVA (*n* = 3, ^∗^*p* < 0.05, ^∗∗^*p* < 0.01).

We found that *E. coli* can cause liver damage in WT mice. Further, we injected the equivalent amount of *E. coli* into the abdominal cavity of CD38^−/−^ and CD38^−/−^TLR4^mut^ mice to observe their response to bacteria. Three hours later, the liver and serum samples were collected for further study. Our results showed that in the liver of CD38-deficient mice, the expression levels of TLR4 mRNA and protein were significantly increased and CD38^−/−^TLR4^mut^ mice showed lower expression levels of TLR4 (Figures 2(a) and 2(b)). And also, the results of serological analysis showed that the concentration of AST in serum of CD38-deficient mice was significantly higher than that of WT mice, while that of CD38^−/−^TLR4^mut^ mice was decreased (Figure 2(c)). Nevertheless, there was no significant difference in serum ALT concentration among the three groups (Figure 2(d)). And the H&E staining results showed that compared with WT mice, CD38-deficient mice turned into having more severe pathological damage (Figures 2(e), 2(f), 2(h), and 2(i)), specifically more severe edema (yellow arrow), inflammatory cell infiltration (blue arrow), punctate necrosis (black arrow), and binucleate hepatocytes (green arrow). And in the liver of CD38^−/−^TLR4^mut^ mice, the pathological changes of the above were obviously relieved (Figures 2(h)–2(k)). These results show that *E. coli* can cause more serious liver injury in CD38^−/−^ mice than in WT mice, and the loss of CD38 will aggravate the damage of bacteria to the liver, but TLR4 mutation can reduce liver injury in septicemia aggravated by CD38 deletion.

### 3.3. Expression of TLR4-NF-*κ*B and Inflammatory Cytokines Increased in the Liver of CD38^−/−^ Mice Infected with *E. coli*

**Figure 3:**
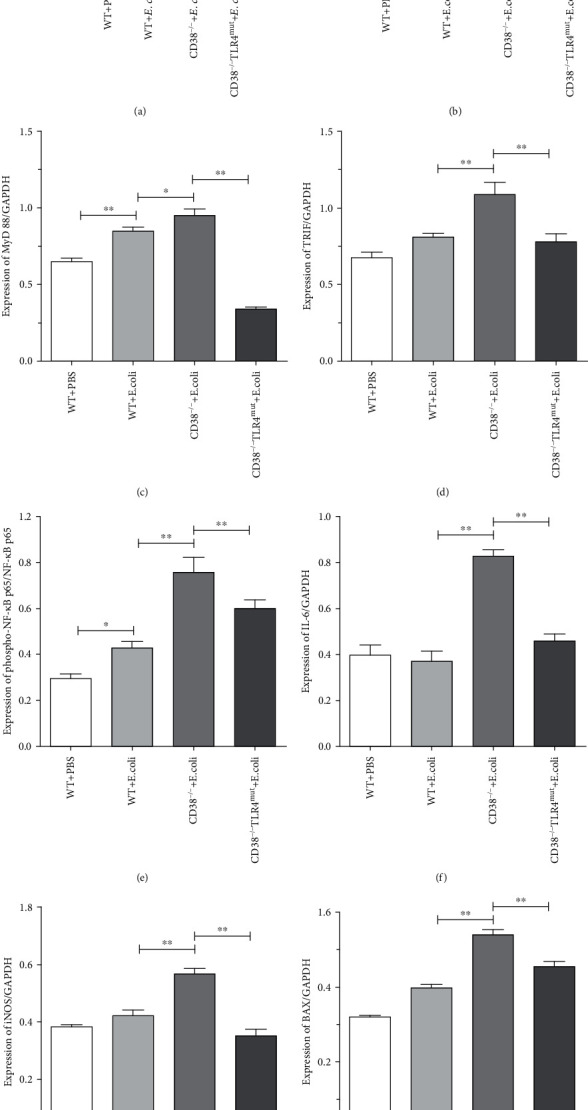
Expression of TLR4-NF-*κ*B and inflammatory cytokines in the liver was detected by Western blot. Changes in expression of liver TLR4 and proinflammatory cytokines in WT, CD38^−/−^, and CD38^−/−^TLR4^mut^ mice were detected at 3 hours after *E. coli* stimulation by Western blot. The expressions of TLR4, MyD88, TRIF, NF-*κ*B p65 and phospho-NF-*κ*B p65, IL-6, iNOS, and BAX were measured (a). And relative levels of TLR4 to GAPDH (b), MyD88 to GAPDH (c), TRIF to GAPDH (d), phospho-NF-*κ*B p65 to NF-*κ*B p65 (e), IL-6 to GAPDH (f), iNOS to GAPDH (g), and BAX to GAPDH (h) were analyzed by ImageJ software. Data are presented as means ± standard deviation. Statistical significance was determined by one-way ANOVA (*n* = 3, ^∗^*p* < 0.05, ^∗∗^*p* < 0.01).

In CD38-deficient mice, we observed more severe liver injury after *E. coli* challenge. Further, after 3 hours of *E. coli* challenge, we extracted the liver proteins of WT and CD38^−/−^ and CD38^−/−^TLR4^mut^ mice and detected the expression of TLR4-NF-*κ*B and inflammatory cytokines to explore potential mechanisms. So Western blot was performed, and the results displayed that under bacterial stimulation, CD38-knockout mice showed more severe inflammatory response than WT mice. In CD38^−/−^ mice, the expression of TLR4 (Figures 3(a) and 3(b)), MyD88 (Figures 3(a) and 3(c)), and TRIF (Figures 3(a) and 3(d)) was significantly higher than that in WT mice, and also, the expression of phosphorylated NF-*κ*B p65 was significantly higher (Figures 3(a) and 3(e)). Furthermore, we also detected the protein levels of IL-6 (Figures 3(a) and 3(f)), iNOS (Figures 3(a) and 3(g)), and BAX (Figures 3(a) and 3(h)). As expected, compared with WT mice, the expression of these inflammatory proteins in the liver of CD38^−/−^ mice increased significantly. However, the expression levels of these inflammatory proteins decreased significantly in the liver of CD38^−/−^TLR4^mut^ mice. The above results manifested that CD38-deficient mice were more susceptible to *E. coli*, and more inflammatory proteins were expressed in the liver, resulting in more severe inflammatory reactions. And the inflammatory reactions upregulated the protein expression of downstream inflammatory factors such as IL-6, iNOS, and BAX through the TLR4-NF-*κ*B p65 pathway.

### 3.4. Bacterial Stimulation Significantly Activated the TLR4-ERK Pathway in CD38^−/−^ Mice

**Figure 4:**
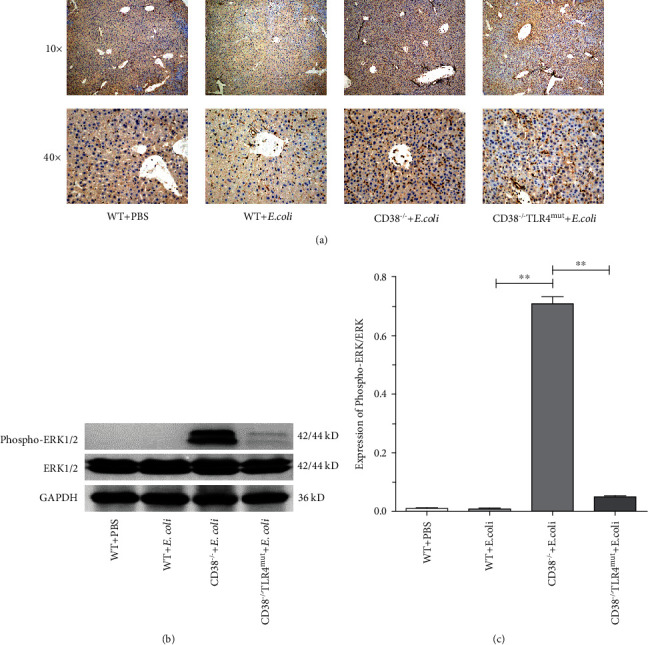
The expression of ERK1/2 was detected in mice induced by *E. coli.* After 3 hours of bacterial stimulation, liver tissues of WT CD38^−/−^ and CD38^−/−^TLR4^mut^ mice were taken out to detect the expression of ERK1/2. The liver tissue was stained by immunohistochemistry, and the pictures were obtained by using an Olympus electron microscope (a). The expression of ERK1/2 was detected by Western blot (b), and relative levels of phospho-ERK1/2 to ERK1/2 (c) were analyzed by ImageJ software. Data are presented as means ± standard deviation. Statistical significance was determined by one-way ANOVA (*n* = 3, ^∗∗^*p* < 0.01).

We found that CD38 deletion stimulated by *E. coli* can aggravate the inflammatory response of mice through the TLR4-NF-*κ*B p65 pathway. Further, we want to explore whether MAPK, also an inflammatory-related signal pathway, is involved in the strong inflammatory response caused by *E. coli* in CD38^−/−^ mice. After 3 hours of *E. coli* stimulation, the liver was taken for immunohistochemical staining. The results showed that compared with the WT PBS control group, bacterial stimulation could increase the nucleus expression of ERK1/2, which means that the phosphorylation level is increased, in liver tissue. And as expected, the expression of ERK1/2-positive cells in liver tissue of CD38^−/−^ mice was significantly higher than that of WT mice (Figure 4(a)). In order to further verify the role of ERK1/2 in inflammatory mediators, we extracted the liver protein of mice for Western blot. The protein band showed that the phosphorylation level of ERK1/2 in the liver of CD38^−/−^ mice was significantly higher than that of WT mice (Figures 4(b) and 4(c)). And interestingly, the results also showed that the activation level of ERK1/2 decreased significantly in CD38^−/−^TLR4^mut^ mice. From the above results, we can know that CD38 deficiency aggravates the inflammatory response of the liver caused by bacterial invasion through the TLR4-ERK1/2 pathway.

### 3.5. CD38 Deficiency Aggravates Liver Injury in Septicemia Mediated by Pyroptosis

**Figure 5:**
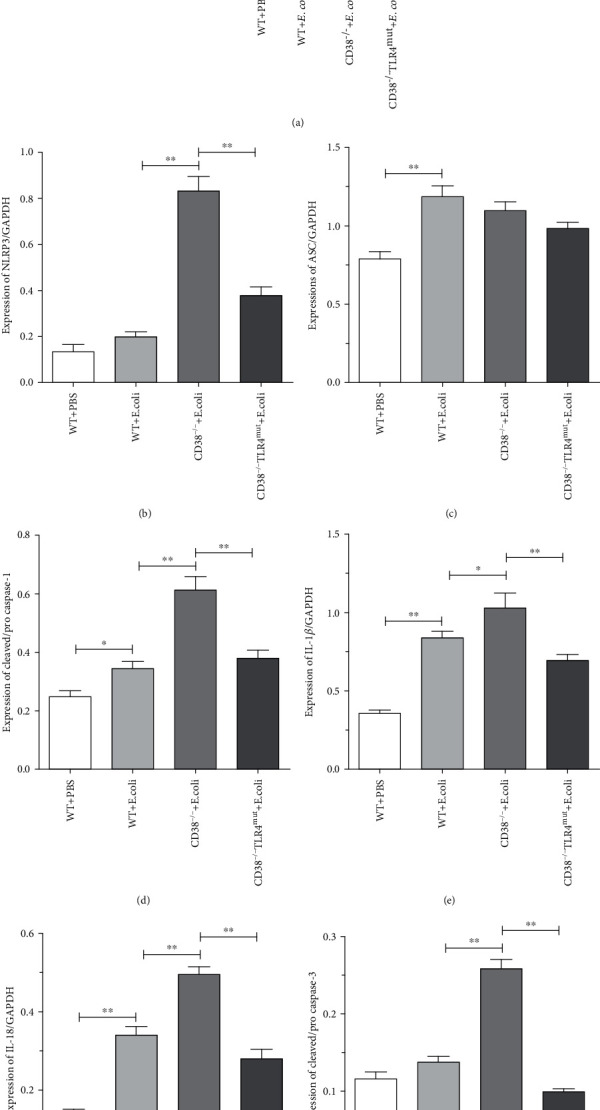
The expression levels of pyroptosis-related markers were detected by Western blot. The expressions of liver pyroptosis proteins in WT, CD38^−/−^, and CD38^−/−^TLR4^mut^ mice were detected at 3 hours after *E. coli* stimulation by Western blot. The expressions of NLRP3, ASC, procaspase-1, cleaved caspase-1, IL-1*β*, IL-18, procaspase-3, and cleaved caspase-3 were measured (a). And relative levels of NLRP3 to GAPDH (b), ASC to GAPDH (c), cleaved to procaspase-1 (d), IL-1*β* to GAPDH (e), IL-18 to GAPDH (f), and cleaved to procaspase-3 (g) were analyzed by ImageJ software. Data are presented as means ± standard deviation. Statistical significance was determined by one-way ANOVA (*n* = 3, ^∗^*p* < 0.05, ^∗∗^*p* < 0.01).

In the previous results, we found that CD38 deletion can lead to more severe sepsis related-liver injury, and there are studies reporting that in sepsis-related liver injury, pyroptosis activates immune cells and hugely induces inflammation [42]. Therefore, we investigated the role of pyroptosis in the liver injury of septicemia aggravated by CD38 deficiency. Western blot was performed to detect the levels of pyroptosis-related markers in liver tissues. And the results of Western blot showed that compared with the PBS control group, bacterial stimulation significantly increased the expression of ASC (Figures 5(a) and 5(c)), IL-1*β* (Figures 5(a) and 5(e)), and IL-18 (Figures 5(a) and 5(f)). In accordance with the expected results, we observed a significant increase in the expression levels of pyroptosis-related markers in the liver of CD38-deficient septicemia mice, such as NLRP3 (Figures 5(a) and 5(b)), cleaved caspase-1 (Figures 5(a) and 5(d)), IL-1*β* (Figures 5(a) and 5(e)), and IL-18 (Figures 5(a) and 5(f)), accompanied by the increase in cleaved caspase-3 (Figures 5(a) and 5(g)). Interestingly, these increased expression levels of pyroptosis-related markers caused by CD38 deletion can be reversed by TLR4 mutations. Taken together, these results suggested that CD38 deletion leads to more severe pyroptosis in bacteria-induced septic liver injury and is associated with the TLR4 signaling pathway.

### 3.6. CD38 Deficiency Aggravates GSDMD-Mediated Pyroptosis in the Liver of Sepsis Mice

**Figure 6:**
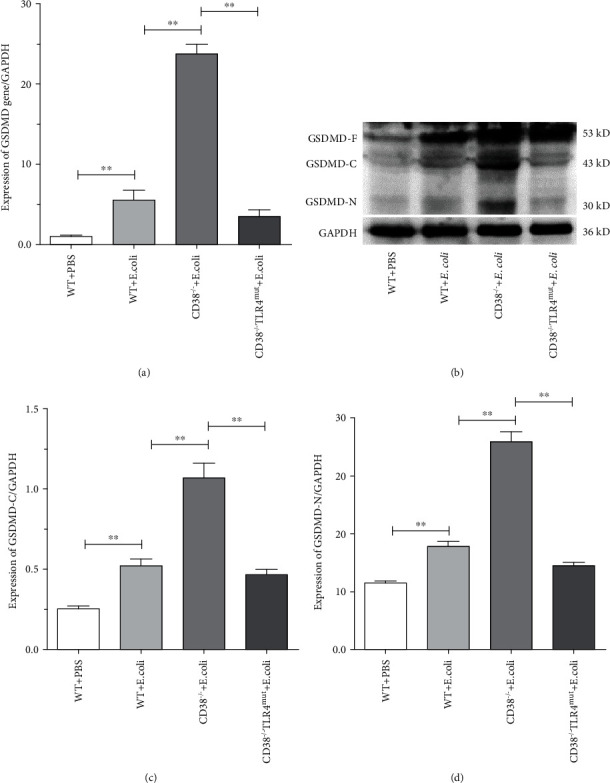
The expression of GSDMD was detected by Western blot. The expressions of GSDMD in the liver of WT, CD38^−/−^, and CD38^−/−^TLR4^mut^ mice were detected at 3 hours after *E. coli* stimulation. The mRNA expression of GSDMD was measured (a) by RT-qPCR. And the expression of GSDMD was detected (b) by Western blot. And relative levels of GSDMD-C to GAPDH (c) and GSDMD-N to GAPDH (d) were analyzed by ImageJ software. Data are presented as means ± standard deviation. Statistical significance was determined by one-way ANOVA (*n* = 3, ^∗∗^*p* < 0.01).

More research reports suggest that GSDMD plays an important role in pyroptosis and is regarded as a pyroptosis executioner [43]. Similarly, we also detected the expression level of GSDMD to observe its role in pyroptosis. The results of RT-qPCR showed that under the stimulation of *E. coli*, the mRNA of GSDMD increased significantly in WT mice, and CD38 deficiency further increased GSDMD expression level significantly (5-fold to WT), but TLR4 mutation significantly decreased (Figure 6(a)). Recently, it is reported that the active form of GSDMD, termed GSDMD-N, was identified to mediate pyroptotic inflammatory cell death in several diseases [44]. Therefore, we decided to detect the protein expression levels of GSDMD and its activated cleavage C-terminal and N-terminal by Western blot. And the results showed that both of GSDMD-C (Figures 6(b) and 6(c)) and GSDMD-N (Figures 6(b) and 6(d)) fragments were overexpressed in CD38^−/−^ mice than in other groups. And also, we found that the mutation of TLR4 in CD38-deficient mice decreased the expression level of cleaved GSDMD significantly. From these results, we can draw a conclusion that TLR4 mutation can mitigate CD38 deficiency which aggravates liver injury in sepsis by GSDMD-mediated pyroptosis.

## 4. Discussion

The role of CD38 in promoting or inhibiting inflammation in the inflammatory response has been controversial, and it plays different roles in the inflammation of different organs. In a mouse model of colitis, CD38 deficiency relieves colonic inflammatory symptoms [18]. Blocking the CD38 pathway protected the hippocampus from apoptosis, oxidative stress, and ultrastructural morphology damage in a septic rat [45], and also, the hearts, livers, and kidneys of septic rats were protected from sepsis-induced damage [8]. On the contrary, accumulated evidence indicates that CD38-knockout (KO) mice are more susceptible to pathogenic bacterial infection [9, 46, 47] and CD38 deficiency can aggravate the inflammatory response [12]. And a research by Lischke et al. showed that CD38-KO mice were highly susceptible to *Listeria monocytogenes* infection and absence of CD38 caused alterations of the migration pattern of neutrophils and inflammatory monocytes to sites of infection and more accumulation of cells in the liver [10]. CD38 overexpression protects against LPS/GalN-induced acute liver injury [17], and CD38-KO mice demonstrated significant increases in serum ALT and AST [16]. In accordance with the results of the latter, we infected WT and CD38^−/−^ mice with *E. coli*. Compared with WT, CD38^−/−^ mice showed to be more susceptible to *E. coli* causing more severe liver injury, more inflammatory cell infiltration, increased expression of inflammatory cytokines, and upregulated expression of the apoptosis gene.

It has been reported that CD38 deletion can activate NF-*κ*B and the expression of downstream inflammatory factors to aggravate the inflammatory response [12], and this pathway is related to TLR4 [48]. Takayashiki et al. showed that increased expression of TLR4 enhances endotoxin-induced hepatic failure [49], and our previous studies have shown that CD38 deficiency enhances TLR4 expression in the kidneys of LPS-induced septic mice [50]. In line with these, in this study, we also found that CD38 deletion can upregulate TLR4 expression, induce phosphorylation of downstream NF-*κ*B p65, and activate the expression of downstream inflammatory factors and apoptosis gene in an *E. coli*-induced liver injury model, and TLR4 mutation significantly rescues the liver injury from inflammatory response aggravated by CD38 deficiency.

Recently, growing evidence suggests that the NLRP3 inflammasome activation is an important regulator of pyroptosis, which plays various roles in the development of liver diseases [26]. There is a research reporting that experiments in cell cultures, mice, and human samples show that a specific form of cell death, called pyroptosis, leads to the release of complex inflammatory particles, the NLRP3 inflammasome, from inside hepatocytes into the extracellular space, and from there, they are taken up by other cells and thereby mediate inflammatory signals [24]. Chen et al. demonstrated that the hepatic cell pyroptosis increased in a time-dependent manner with the highest rate at 24 h after CLP-induced sepsis and also the severity of liver pyroptosis was correlated with the liver damage [51]. Inhibiting the liver pyroptosis by NLRP3 and caspase-1 inhibitors could reduce the degree of septic acute liver injury [28] and knockdown of NLRP3 or GSDMD significantly restored LPS/D-Gal-induced acute liver injury and lethality [52]. Moreover, our previous study [53] showed that CD38 deletion upregulates TLR4 expression, and also, there is a paper [54] reporting that CD38 can induce inflammasome-mediated activation of caspase-1 by activating NLRP3 in head and neck squamous cell carcinoma (HNSCC). And in this study, we observed severe pyroptosis and serious damage of the liver in septicemia-related liver injury in CD38-deficient mice, whereas both pyroptosis and damage of the liver can be significantly reversed in TLR4 mutant mice, accompanied by the decrease in NLRP3 and GSDMD. Therefore, we concluded that the CD38-regulated TLR4-NLRP3-GSDMD pathway plays an important role in liver injury of septicemia and its mechanism needs further study.

As early as 2001, Guha and Mackman had reported that LPS stimulates the activation of various MAPK pathways with the signaling receptor TLR4, and specific inhibitors of the ERK1/2 and p38 MAPK pathways blocked nuclear NF-*κ*B activity and the transactivation activity of NF-*κ*B p65 [55]. And in particular, studies have demonstrated that the ERK1/2 signaling pathway participates in the mitochondrial dysfunction, oxidative stress, cell apoptosis, and inflammation [37, 56]. Recently, there are research reports that in an acetaminophen- (APAP-) induced liver injury mouse model, ERK1/2 activity was markedly increased in the APAP group than in the control group, and no significant difference was observed in the hepatic JNK and p38 protein expression and phosphorylation [34, 38], indicating that ERK1/2 is more related to liver injury than another two members of MAPKs. Similarly, our results showed that ERK1/2 nuclear translocation and phosphorylation levels in the lesion liver of CD38^−/−^ mice were significantly higher than those of WT mice after *E. coli* stimulation. In addition, we also observed that NF-*κ*B p65 activation increased and the expression of inflammatory factors and apoptosis genes was significantly upregulated. These results strongly suggested that ERK1/2 is involved in the occurrence and development of bacterial liver injury and that CD38 deletion can activate the ERK1/2 signaling pathway to aggravate liver injury. The same as the current data, a report showed that the level of ERK1/2 phosphorylation in liver and lung tissue increased significantly compared to that in sham in polymicrobial sepsis; activated ERK1/2 can induce the activation of NF-*κ*B and a series of proinflammatory cytokine gene expressions, leading to systemic inflammatory response and even multiple organ dysfunction in sepsis [57].

In conclusion, the current study suggested that *E. coli* can enter the liver tissue and cause bacterial liver injury, which is manifested by histopathological changes, liver function damage, intrahepatic inflammation, and pyroptosis increase. Importantly, the above-mentioned reactions are more serious in the liver of CD38^−/−^ mice. At the same time, TLR4-NF-*κ*B p65, ERK1/2 phosphorylation, and pyroptosis-related marker proteins are significantly increased, aggravating the liver damage, while TLR4 mutation can significantly reduce the above reaction and improve the liver injury caused by bacteria. Our results showed that CD38 deficiency causes severe bacterial liver injury through TLR4-NLRP3-GSDMD-mediated pyroptosis. This study also shows that CD38-deficient host is susceptible to bacteria, which will aggravate the damage caused by bacterial invasion.

## 5. Conclusions

Based on the results of this study, we concluded that CD38 deficiency could increase *E. coli*-induced inflammatory response and activate TLR4-NLRP3-GSDMD-mediated pyroptosis, aggravating liver injury in septic mice. Thus, TLR4 inhibitors or CD38 activators may serve as potential drugs to attenuate *E. coli*-induced liver injury in septicemia.

## Data Availability

The data used to support the findings of this study are available from the corresponding author upon request.
